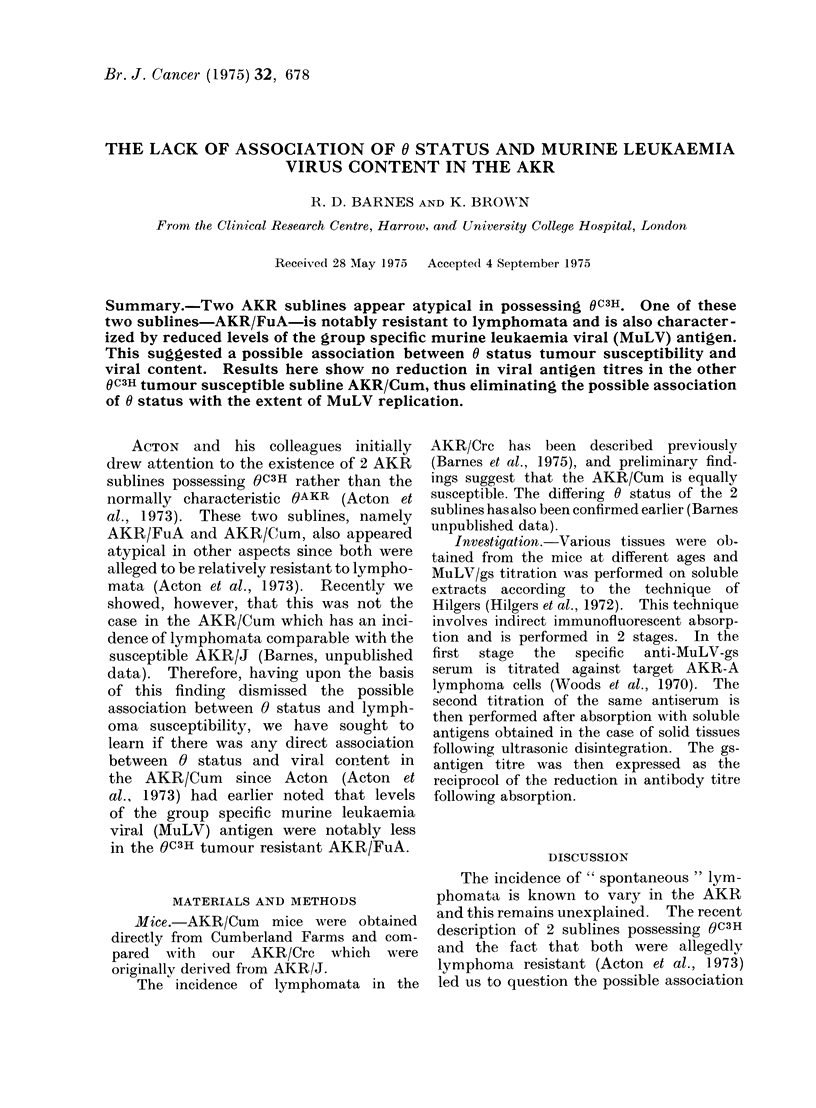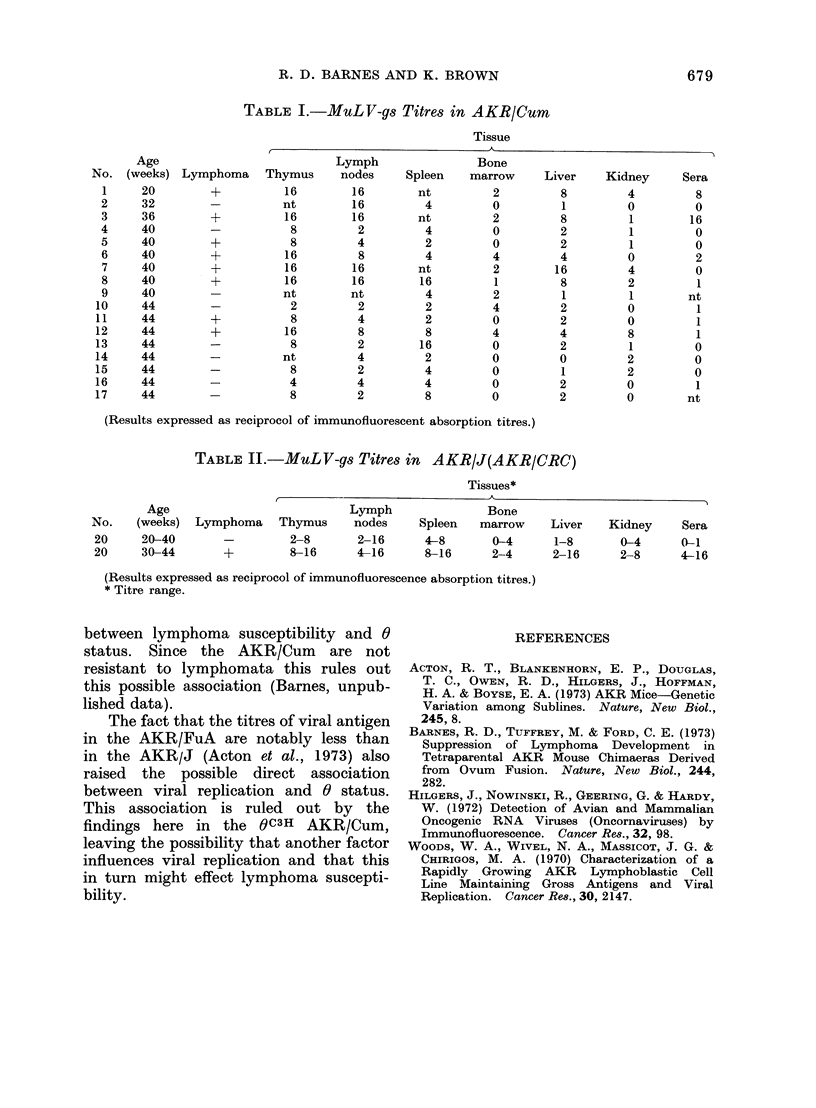# The lack of association of theta status and murine leukaemia virus content in the AKR.

**DOI:** 10.1038/bjc.1975.278

**Published:** 1975-12

**Authors:** R. D. Barnes, K. Brown

## Abstract

Two AKR sublines appear atypical in possessing theta C3H. One of these two sublines - AKR/FuA - is notably resistant to lymphomata and is also characterized by reduced levels of the group specific murine leukaemia viral (MuLV) antigen. This suggested a possible association between theta status tumour susceptibility and viral content. Results here show no reduction in viral antigen titres in the other theta C3H tumour susceptible subline AKR/Cum, thus eliminating the possible association of theta status with the extent of MuLV replication.


*Br. J. Cancer* (1975) **32**, 678

### THE LACK OF ASSOCIATION OF $\theta$ STATUS AND MURINE LEUKAEMIA VIRUS CONTENT IN THE AKR

R. D. BARNES AND K. BROWN

*From the Clinical Research Centre, Harrow, and University College Hospital, London*

Received 28 May 1975 Accepted 4 September 1975

**Summary.—Two AKR sublines appear atypical in possessing $\theta^{C3H}$. One of these two sublines—AKR/FuA—is notably resistant to lymphomata and is also characterized by reduced levels of the group specific murine leukaemia viral (MuLV) antigen. This suggested a possible association between $\theta$ status tumour susceptibility and viral content. Results here show no reduction in viral antigen titres in the other $\theta^{C3H}$ tumour susceptible subline AKR/Cum, thus eliminating the possible association of $\theta$ status with the extent of MuLV replication.**

ACTON and his colleagues initially drew attention to the existence of 2 AKR sublines possessing $\theta^{C3H}$ rather than the normally characteristic $\theta^{AKR}$ (Acton *et al.*, 1973). These two sublines, namely AKR/FuA and AKR/Cum, also appeared atypical in other aspects since both were alleged to be relatively resistant to lymphomata (Acton *et al.*, 1973). Recently we showed, however, that this was not the case in the AKR/Cum which has an incidence of lymphomata comparable with the susceptible AKR/J (Barnes, unpublished data). Therefore, having upon the basis of this finding dismissed the possible association between $\theta$ status and lymphoma susceptibility, we have sought to learn if there was any direct association between $\theta$ status and viral content in the AKR/Cum since Acton (Acton *et al.*, 1973) had earlier noted that levels of the group specific murine leukaemia viral (MuLV) antigen were notably less in the $\theta^{C3H}$ tumour resistant AKR/FuA.

### MATERIALS AND METHODS

*Mice.*—AKR/Cum mice were obtained directly from Cumberland Farms and compared with our AKR/Crc which were originally derived from AKR/J.

The incidence of lymphomata in the AKR/Crc has been described previously (Barnes *et al.*, 1975), and preliminary findings suggest that the AKR/Cum is equally susceptible. The differing $\theta$ status of the 2 sublines has also been confirmed earlier (Barnes unpublished data).

*Investigation.*—Various tissues were obtained from the mice at different ages and MuLV/gs titration was performed on soluble extracts according to the technique of Hilgers (Hilgers *et al.*, 1972). This technique involves indirect immunofluorescent absorption and is performed in 2 stages. In the first stage the specific anti-MuLV-gs serum is titrated against target AKR-A lymphoma cells (Woods *et al.*, 1970). The second titration of the same antiserum is then performed after absorption with soluble antigens obtained in the case of solid tissues following ultrasonic disintegration. The gs-antigen titre was then expressed as the reciprocol of the reduction in antibody titre following absorption.

### DISCUSSION

The incidence of " spontaneous " lymphomata is known to vary in the AKR and this remains unexplained. The recent description of 2 sublines possessing $\theta^{C3H}$ and the fact that both were allegedly lymphoma resistant (Acton *et al.*, 1973) led us to question the possible association

TABLE I.—*MuLV-gs Titres in AKR/Cum*

| | | | Tissue | | | | | | |
|---|---|---|---|---|---|---|---|---|---|
| No. | Age (weeks) | Lymphoma | Thymus | Lymph nodes | Spleen | Bone marrow | Liver | Kidney | Sera |
| 1 | 20 | + | 16 | 16 | nt | 2 | 8 | 4 | 8 |
| 2 | 32 | — | nt | 16 | 4 | 0 | 1 | 0 | 0 |
| 3 | 36 | + | 16 | 16 | nt | 2 | 8 | 1 | 16 |
| 4 | 40 | — | 8 | 2 | 4 | 0 | 2 | 1 | 0 |
| 5 | 40 | + | 8 | 4 | 2 | 0 | 2 | 1 | 0 |
| 6 | 40 | + | 16 | 8 | 4 | 4 | 4 | 0 | 2 |
| 7 | 40 | + | 16 | 16 | nt | 2 | 16 | 4 | 0 |
| 8 | 40 | + | 16 | 16 | 16 | 1 | 8 | 2 | 1 |
| 9 | 40 | — | nt | nt | 4 | 2 | 1 | 1 | nt |
| 10 | 44 | — | 2 | 2 | 2 | 4 | 2 | 0 | 1 |
| 11 | 44 | + | 8 | 4 | 2 | 0 | 2 | 0 | 1 |
| 12 | 44 | + | 16 | 8 | 8 | 4 | 4 | 8 | 1 |
| 13 | 44 | — | 8 | 2 | 16 | 0 | 2 | 1 | 0 |
| 14 | 44 | — | nt | 4 | 2 | 0 | 0 | 2 | 0 |
| 15 | 44 | — | 8 | 2 | 4 | 0 | 1 | 2 | 0 |
| 16 | 44 | — | 4 | 4 | 4 | 0 | 2 | 0 | 1 |
| 17 | 44 | — | 8 | 2 | 8 | 0 | 2 | 0 | nt |

(Results expressed as reciprocol of immunofluorescent absorption titres.)

TABLE II.—*MuLV-gs Titres in AKR/J(AKR/CRC)*

| | | | Tissues* | | | | | | |
|---|---|---|---|---|---|---|---|---|---|
| No. | Age (weeks) | Lymphoma | Thymus | Lymph nodes | Spleen | Bone marrow | Liver | Kidney | Sera |
| 20 | 20–40 | — | 2–8 | 2–16 | 4–8 | 0–4 | 1–8 | 0–4 | 0–1 |
| 20 | 30–44 | + | 8–16 | 4–16 | 8–16 | 2–4 | 2–16 | 2–8 | 4–16 |

(Results expressed as reciprocol of immunofluorescence absorption titres.)
* Titre range.

between lymphoma susceptibility and $\theta$ status. Since the AKR/Cum are not resistant to lymphomata this rules out this possible association (Barnes, unpublished data).

The fact that the titres of viral antigen in the AKR/FuA are notably less than in the AKR/J (Acton *et al.*, 1973) also raised the possible direct association between viral replication and $\theta$ status. This association is ruled out by the findings here in the $\theta^{C3H}$ AKR/Cum, leaving the possibility that another factor influences viral replication and that this in turn might effect lymphoma susceptibility.

### REFERENCES

ACTON, R. T., BLANKENHORN, E. P., DOUGLAS, T. C., OWEN, R. D., HILGERS, J., HOFFMAN, H. A. & BOYSE, E. A. (1973) AKR Mice—Genetic Variation among Sublines. *Nature, New Biol.*, **245**, 8.

BARNES, R. D., TUFFREY, M. & FORD, C. E. (1973) Suppression of Lymphoma Development in Tetraparental AKR Mouse Chimaeras Derived from Ovum Fusion. *Nature, New Biol.*, **244**, 282.

HILGERS, J., NOWINSKI, R., GEERING, G. & HARDY, W. (1972) Detection of Avian and Mammalian Oncogenic RNA Viruses (Oncornaviruses) by Immunofluorescence. *Cancer Res.*, **32**, 98.

WOODS, W. A., WIVEL, N. A., MASSICOT, J. G. & CHIRIGOS, M. A. (1970) Characterization of a Rapidly Growing AKR Lymphoblastic Cell Line Maintaining Gross Antigens and Viral Replication. *Cancer Res.*, **30**, 2147.